# PedGenie: an analysis approach for genetic association testing in extended pedigrees and genealogies of arbitrary size

**DOI:** 10.1186/1471-2105-7-209

**Published:** 2006-04-18

**Authors:** Kristina Allen-Brady, Jathine Wong, Nicola J Camp

**Affiliations:** 1 Genetic Epidemiology Division, Department of Medical Informatics, University of Utah Salt Lake City, Utah, USA

## Abstract

**Background:**

We present a general approach to perform association analyses in pedigrees of arbitrary size and structure, which also allows for a mixture of pedigree members and independent individuals to be analyzed together, to test genetic markers and qualitative or quantitative traits. Our software, PedGenie, uses Monte Carlo significance testing to provide a valid test for related individuals that can be applied to any test statistic, including transmission disequilibrium statistics. Single locus at a time, composite genotype tests, and haplotype analyses may all be performed. We illustrate the validity and functionality of PedGenie using simulated and real data sets. For the real data set, we evaluated the role of two tagging-single nucleotide polymorphisms (tSNPs) in the DNA repair gene, *NBS1*, and their association with female breast cancer in 462 cases and 572 controls selected to be *BRCA1/2 *mutation negative from 139 high-risk Utah breast cancer families.

**Results:**

The results from PedGenie were shown to be valid both for accurate *p*-value calculations and consideration of pedigree structure in the simulated data set. A nominally significant association with breast cancer was observed with the *NBS1 *tSNP rs709816 for carriage of the rare allele (OR = 1.61, 95% CI = 1.10–2.35, p = 0.019).

**Conclusion:**

PedGenie is a flexible and valid statistical tool that is intuitively simple to understand, makes efficient use of all the data available from pedigrees without requiring trimming, and is flexible to the types of tests to which it can be applied. Further, our analyses of real data indicate *NBS1 *may play a role in the genetic etiology of heritable breast cancer.

## Background

Family-based tests of allelic association have received much attention recently due in part to their robustness against a bias that may arise in population data. Population based association studies may be hindered by genetic stratification that exists even within relatively homogeneous populations [1,2], resulting in spurious associations that markedly increase with sample size [3,4]. Family-based association studies that focus on the preferential transmission from parents to affected offspring are robust to the effects of population stratification, admixture, and non-random mating [5,6] and hence avoid the bias of population stratification. Additional advantages of family-based designs include error checking of genotype data, the capacity to detect parent-of-origin effects, and the improved efficiency and cost effectiveness of being able to perform association studies on cases previously ascertained for linkage studies [7].

Use of family data for allelic association testing includes the additional challenge of accounting for correlations between related individuals. Without correction for the genetic dependence of related individuals, an underestimate of the variance of a desired statistic and an increase in type I error will result [8]. A number of family-based association tests that appropriately account for correlations between related individuals have been developed, many of which are extensions of the transmission disequilibrium test (TDT) (See review of family based methods [9]). The TDT was originally proposed as a test for linkage disequilibrium in family trios, consisting of two parents (at least one heterozygous) and an affected offspring [10]. The TDT has been extended to allow for the use of siblings instead of parents, multiallelic markers, and extensions to quantitative traits and covariates (see review [11]). Most TDT based tests, however, are limited, as they focus on only small nuclear families and/or a particular genotype configuration to be valid tests.

Extensions of traditional case-control association and transmission disequilibrium tests to multi-generational pedigrees also exist [12-22]. However, many of these methods restrict analyses to either dichotomous dependent variables[12,13,20,22] or quantitative traits [14,16,21,23]. For those methods that analyze quantitative traits, some require the trait to be normally distributed [14,16,23]. Others limit the size of a pedigree that can be studied because they require calculation of inherited by descent (IBD) parameters [14,16,20]; although the pedigree size for which IBD can be determined is increasing [24], pedigree size, for particularly large extended pedigrees or genealogical resources, still remains a limiting factor. Other methods decompose large pedigrees into nuclear families [4,12,13,15,19]; if these methods are applied to a small sample of large pedigrees, this will lead to a substantial loss of information and power, and may risk inflation of false positive results.

A number of large and sometimes complex genealogy-based populations are being ascertained to study complex traits, including the Hutterites, the Pima Indians, Sardinia isolates, as well as studies in Iceland and Utah. Utilizing all cases in a pedigree or genealogy for an association study rather than just one case or nuclear family per pedigree (if one adopts an 'independence' protocol) increases power by increasing the effective sample size [8]. Ideally, analyses utilizing all available information on pedigree structure are most informative and efficient.

We previously proposed a method that could perform valid simple case-control association analyses on extended pedigrees of arbitrary size and structure using an empirical approach [8]. Here, we present an expanded more versatile software tool, PedGenie that incorporates a number of common association and transmission disequilibrium statistics for qualitative and quantitative data. PedGenie accounts for correlations between related individuals, based on the original pedigree structure. A Monte Carlo approach is employed to generate an empirical null distribution from which significance for a statistic of interest can be determined. Pedigrees of arbitrary size or structure, from singletons (single, independent individuals) to entire genealogies with loops can all be analyzed simultaneously. Furthermore, our method is able to analyze multi-locus data either as composite genotypes (multiple insult hypothesis, where phase is not considered) and haplotypes (where phase is important), assuming that the correct haplotypes have been assigned to pedigree members. We illustrate the validity and utility of PedGenie through its application to simulated data as well as test the association between two tagging-SNPs in the DNA repair gene, *NBS1*, and breast cancer.

## Implementation

Our software tool PedGenie, which requires Java 1.5 for execution, is freely available (see Availability and requirements). We begin with a description of its functionality for inheritance of a biallelic marker, although all analyses can be performed with multi-allelic markers, followed by an explanation of how PedGenie handles haplotype data. Assume that we have a resource of large extended pedigrees containing cases with a particular disease. Although the genealogy of individuals may be known, typically genotype and perhaps phenotype data will be available only on individuals near the bottom of the pedigree, yet relationships between individuals are still known. Under the null hypothesis of no association, the genotype of an affected individual in the pedigree is independent of their disease status. Based on this null hypothesis, new genotypes for individuals in the pedigree may be generated, which we term a "null genotypic configuration"; both relationships within the pedigree and the missing data structure are maintained in the null genotypic configuration. New traits, however, are not generated for individuals, thus also maintaining the original phenotypic familial correlations. Through generating multiple null genotypic configurations and calculating a statistic of interest for each, an empirical null distribution can be created for the statistic and this null distribution can be used to determine significance of an observed statistic (calculated from the real genotypic data).

An outline of steps for PedGenie is as follows. First, allele frequencies for the markers of interest are estimated from the data. These can be estimated by four different methods: genotyped founders only, all genotyped individuals, all founders with statistically inferred genotypes and user-defined. For the first option, allele frequency estimation is made using data from genotyped founders only, hence it is representative of the general population from which the study sample was obtained but requires that many of the founders have genotype data available. The second option uses all genotyped individuals, ignoring dependence between relatives, for which the point estimate is unbiased [25]. The third option statistically infers the founder genotypes using maximum likelihood estimation, and then calculates allele frequencies from the inferred (or actual, if observed) genotypes of all the founders [26]. We recommend statistically inferring founder genotypes if there are a small to moderate number of relatively large pedigrees. If there are a large number of relatively small pedigrees and the number of genotyped founders in the resource is limited, we recommend the 'all' option, which is the current default in PedGenie. The final option allows a user to enter allele frequencies.

Second, alleles are assigned to the pedigree founders randomly, but in proportion to these allele frequencies, and a Mendelian gene-drop is performed. That is, the gene-drop is performed independently of trait information. For example, alleles A and Bwill be transmitted from a parent with genotype AB to their children with equal probability. The resulting null genotype configuration on each pedigree, therefore represents a possible configuration under the null hypothesis of no association between allele and disease. Assignment of alleles randomly to pedigree founders, who are assumed to be independent individuals in the population of interest, will inherently be in Hardy-Weinberg equilibrium.

Third, the statistic of interest is calculated using the null genotype configuration and the true phenotype data, which we term S_*i*_. The S_*i *_statistic is from the null distribution since it was derived from data simulated under the null hypothesis. Steps 2 and 3 are repeated multiple (N) times, and the series of null statistics stored. Hence an empirical null distribution is created for the statistic of interest, conditional on the particular pedigree and phenotype structures contained in the resource.

Finally, the observed statistic, S_0_, is computed based on the true genotype and phenotype data using the same statistic of interest. This observed statistic is then compared to the empirical null distribution to determine significance as follows:

*p *= ∑ [I(S_*i*_)]/N for *i *= 1 to N

where: I(S_*i*_) = 1 if S_*i *_≥ S_0_

    0 otherwise

The specified null hypothesis is rejected if the *p*-value is less than or equal to the required level of type I error (α). The accuracy of the empirical *p*-value increases with the size of the empirical null distribution simulated. An N of 2,000 gives a 95% confidence interval around α = 0.05 with a width of 0.02 under the null hypothesis. It is possible that the statistic of interest cannot be calculated, such as when the data are too sparse (e.g., an inability to calculate an odds ratio due to a zero count in a contingency table). PedGenie provides information on the number of simulations for which a statistic can be calculated. If the number of calculated statistics is less than the total number of simulations (i.e., N), this would suggest sparse data for that particular analysis and caution is advised when interpreting the results.

To match the information content of the real data to that of the simulated data, we limit calculation of the statistic of interest in the simulated data to only those individuals with genotype data in the observed sample. In the gene-drop procedure, genotype information is initially simulated for all members of a pedigree; however, those individuals for whom observed genotypic data were not available for a specific locus are reset to missing. In this way, the missing data structure is captured.

### Composite genotype and haplotype-based analyses

Testing multiple loci simultaneously either as composite genotypes or haplotypes is similar to that described above with a few exceptions. For both the composite genotype and haplotype analyses, haplotype frequencies are entered in place of allele frequencies for the gene drop. The gene drop proceeds as above, except haplotypes rather than alleles are dropped through the pedigrees. PedGenie allows the user to enter recombination rates (i.e., θ) between markers and these values are used to determine recombinant events for generation of the empirical null distribution. For composite genotype testing and haplotype testing, user-defined population haplotype frequency estimates are required. The difference between composite genotype and haplotype analyses is that phase information on the observed genotype data is required for the haplotype analysis but is not required for the composite genotype analysis. For haplotypes, PedGenie expects the pedigree genotype data to be ordered. A number of pedigree based haplotype methods are being developed [27-32]. However, none of the haplotype methods that are currently available are able to provide both haplotype frequencies and individual haplotype assignment on large pedigrees with large amounts of missing data, and linkage disequilibrium between multiple markers (see also [33]); hence additional work in this area is required.

### Statistics

Our approach is general, such that an empirical null distribution may be computed for any statistic of interest. PedGenie version 1.2 currently incorporates statistics for both dichotomous and quantitative data outcomes. For data involving a dichotomous variable outcome, the implemented statistics are: basic genotype-based or allele-based Chi-square statistics (for arbitrary number of categories that are user-specified), an odds ratio statistic both for allele counts and genotype data (with 95% confidence intervals determined from the empirical distribution), and a Chi-square trend statistic with user-defined weights. In addition to these classical tests of association, our method is also able to test transmission disequilibrium on large extended pedigrees, including the TDT statistic for trios [10], sibships [34], and combined trios/sibships [34]. For quantitative dependent variable outcomes, a standardized difference in means statistic and an overall analysis of variance (ANOVA) are implemented. PedGenie also can test quantitative TDT statistics based on methods of Allison (TDT_Q5_) [23], Rabinowitz [35], and Monks [11]. Covariate data can be incorporated into these quantitative TDT models. The quantitative TDT statistics are made available by interfacing PedGenie with the freely available QTDT software [36]. In brief, the real data and each simulated genotypic null distribution are communicated to the QTDT package, and results are parsed and summarized over all simulations within PedGenie and an empirical *p*-value is calculated as defined above.

For the traditional case-control analyses (e.g., chi-square, ANOVA, etc.) included in PedGenie, the null hypothesis of no association only is tested. Null genotypic configurations are constructed by assigning genotypes to founders and to descendents, via the Mendelian gene-drop, independent of disease status.

For the transmission disequilibrium statistics in PedGenie, the composite null hypothesis of "no linkage and no association" is tested. This is the mathematical '*inclusive or' *statement, such that the composite null hypothesis (δ (1 – 2θ) = 0) is satisfied under three scenarios: δ = 0 *and *(1 – 2θ) ≠ 0; δ ≠ 0 *and *(1 – 2θ) = 0; or δ = 0 *and *(1 – 2θ) = 0. PedGenie generates the null hypothesis from the third of these. As above, the null hypothesis of no association is constructed by assigning genotypes to founders and to descendents via the Mendelian gene-drop, which assignment is independent of disease status. The null hypothesis of no linkage is satisfied as the transmission of an allele from parent to offspring is generated independent of disease status. The adequacy of generating a null distribution by sampling from the composite null hypothesis of 'no linkage and no association' is illustrated by the fact that with 2,000 simulations PedGenie estimates p-values accurate to the 3^rd ^decimal place for the TDT, combined TDT and quantitative TDT (see Table 1).

The TDT statistics within PedGenie are constructed precisely as presented by the original authors [10,23,34,35]. PedGenie is merely used to provide valid significance levels, which account for the relatedness of multiple trios or sibships in a pedigree. Hence, the robustness to population stratification inherent in those transmission disequilibrium statistics remains. However, with the case-control statistics PedGenie will not protect against population stratification bias if heterogeneous populations are present in the data set.

### Defining genotype, allele, and haplotype groups

PedGenie has been developed to be general with respect to how genotypes, alleles, combinations of genotypes, or haplotypes are grouped together for an analysis. In the simplest case, PedGenie is able to analyze data by a single locus at a time approach, comparing for example, in a biallelic system, carriers of a rare allele '2' (genotype = 2/2 or 1/2 or 2/1) to individuals with the wild type genotype (1/1). User-defined weights can be assigned to these genotype groups and analyzed using any of the above listed statistical tests. The number of separate groups compared is user-defined. Similarly, multiple alleles may be considered as the unit for analysis and grouped together as necessary. For example, in a multi-allelic system, allele 1 could be a group, allele 2 could be another group, and all other alleles could be considered a third group. For composite genotype tests (involving genotypes at multiple loci), PedGenie allows the user to define groups based on genotypes across multiple loci. For example, a user may wish to analyze individuals who are carriers of a rare allele at locus 1 and homozygous at locus 2 compared to individuals with all other genotype combinations. For haplotype-based tests, analogous to the allele tests, the user can define multiple haplotypes in a single group and compare this group to other groups of haplotypes. Similarly to the single locus case, the number of groups to be compared remains user-defined.

Single locus genotype, composite genotype, and haplotypes may be tested using any of the statistics available, including TDT, sibTDT, and the combined TDT for dichotomous traits. However, QTDT analyses are limited to the capabilities of the QTDT package.

### Functionality of PedGenie

We illustrate the validity and functionality of PedGenie using three different data sets. The first two data sets are simulated data to demonstrate that the techniques employed by PedGenie are valid and robust. In the final data set, we illustrate PedGenie's functionality and ability to handle large, extended pedigrees with real data for breast cancer and two tagging-SNPs in the *NBS1 *gene.

#### Validation of statistics with simulated independent data

The purpose of this first data set was simply to illustrate that in set of independent individuals and a set of independent nuclear families that PedGenie computes an empirical *p*-value that corresponds to those from the appropriate standard statistical distribution. The standard distribution is defined as the distribution that typically would be used for a particular test (e.g., the standard distribution for the Chi-square statistical test is the Chi-square distribution).

In the first data set, we simulated data for a biallelic marker with a minor allele frequency of 0.2. We generated a set of 2,000 independent cases and 2,000 independent controls as well as 4,000 independent nuclear families with two offspring. For the nuclear families, we assumed that the two parents were independent and the offspring, one of whom was affected, inherited alleles in a Mendelian fashion. No association between the biallelic marker and affected status was simulated. We also generated a quantitative trait for all individuals that was randomly assigned and normally distributed with a mean of 50 and a standard deviation of 10. The 2,000 independent case and control data were used to validate the Chi-square, Chi-squared trend, odds ratio, the standardized difference in means statistic and ANOVA. The 4,000 independent nuclear family data were used to test the TDT, sib-TDT, combined trio/sib TDT, and the combined trio/sib QTDT method by Monks [11]. Each of these validations was run 1,000 times on PedGenie using a different initial random number seed. For each PedGenie analysis, the empirical null distribution and *p*-value were determined from a sample size of 2,000 null configurations and the allele frequency estimation method 'all'.

#### Validation of inheritance with simulated pedigree data

The second data set was to illustrate that PedGenie appropriately accounts for relationships within a pedigree structure when performing the gene drop. We used simulated data obtained from the 12^th ^Genetic Analysis Workshop (GAW12) [37] and compared empirical *p*-values from PedGenie to an exact pedigree-based method proposed by Slager and Schaid [20] based on the Armitage trend association statistic [38].

The Slager and Schaid method based on the Armitage test for trend [20] accounts for relatedness of individuals by measuring a trend in proportions according to a general measure of genetic dosage, **x**, where **x **is a vector of weights for each genotype. The Armitage test for trend degenerates to the standard Chi-squared test for independence assuming a dominant or recessive mode of inheritance when **x **= (0, 1, 1) or **x **= (0, 0, 1), respectively, where the three indices in the vector **x **represent the wild type, heterozygote and homozygote genotypes. The Slager and Schaid method, in brief, accounts for correlations between relatives by correcting the variance estimate using a correlation matrix that is a function of posterior inherited by descent (IBD) sharing probabilities, estimated by GENEHUNTER [39]. However, prior probabilities using kinship coefficients may also be substituted into the correlation matrix. In this analysis, we compared PedGenie to the Slager and Schaid method incorporating prior probabilities, as these are the probabilities that are sampled using a Mendelian gene-drop.

The GAW12 simulated dataset provided complete phenotypic and genotypic data for 23 extended pedigrees of 1,000 living individuals. Complex relationships between covariate data (quantitative traits, a disease liability, and age-at-onset) and gene sequence variants were also simulated [37]. We selected a single replicate (Replicate 42) and analyzed the association of all variants with a minor allele frequency ≥ 0.01 in the *MG1 *gene and Q1, a quantitative trait; twenty-four percent of the variance of Q1 was attributable to *MG1*. The effects of other covariates, including age, sex, and an environmental component (EF1) were regressed out of Q1 using linear regression prior to the analyses. Individuals in the top tertile (333 individuals) for the residual genetic component of Q1 were designated cases, and those in the bottom tertile (N = 333) controls. For these analyses, we considered a dominant and recessive mode of inheritance for each variant studied. We compared results from PedGenie using the Chi-Square trend statistic, with weights designed to test a dominant and recessive model, to the Slager and Schaid exact method using prior IBD probabilities in the correlation matrix. The empirical null distribution and *p*-value were determined from a sample size of 2,000 null configurations and the allele frequency estimation method 'all'.

#### Testing of the NBS1 gene and breast cancer

The functionality and ability of PedGenie to handle large extended pedigrees was illustrated using individuals selected from 139 Utah high-risk breast cancer pedigrees of Northern and Western European descent, with family size ranging from 1 (a single individual) to 1,195 individuals in a single pedigree; however, typically only individuals at the bottom of each pedigree were genotyped. Individuals were selected and considered high-risk because they belonged to pedigrees with rates of breast cancer exceeding the population rate. This was determined using the Utah Population Database, a database linking genealogy data to the Utah Cancer Registry (UCR)[40] Breast cancer cases were selected to be most likely not attributable to a *BRCA1/2 *mutation, because either the breast cancer case themselves or other family members tested negative for a *BRCA1/2 *mutation, or the family had a low probability of a *BRCA1/2 *mutation based on the number of breast cancer cases present and/or ages at diagnosis of breast cancer within the family. Breast cancer diagnosis information was obtained from either medical records for the subjects or the UCR. All breast cancer cases in the state of Utah must be reported to the UCR by law, thus the UCR is a reliable information source.

Previously we characterized the linkage disequilibrium (LD) structure and identified two tagging-SNPs (tSNPs) that capture 93.8% of the intragenic variation across the *NBS1 *gene [41], and these two tSNPs have been genotyped on our entire study population (see [41] for genotyping details). The two tSNPs used for this study were rs12680687 (minor allele frequency = 0.28) and rs709816 (minor allele frequency = 0.45). For genotype quality control, six individuals were duplicated across all plates and quality control samples were required to have matching genotype assignments. Where possible, Mendelian inheritance was verified; samples with inheritance incompatibilities were either re-genotyped and/or set to missing if they could not be resolved.

From the 139 breast cancer pedigrees, we defined two cohorts: a Case-Control cohort and a Nuclear Family cohort. The Case-Control cohort illustrates PedGenie's ability to perform standard association-based tests, while the Nuclear Family cohort shows the transmission disequilibrium statistics. The Case-Control cohort was composed of 236 breast cancer cases matched to 236 controls based on birth year (within five years), female gender, and age-at-diagnosis, such that the control was cancer free at the age the case was diagnosed. The matched controls were also chosen from the breast cancer pedigree resource and were selected to be as distantly related to any other case or control as possible to increase power, and as old as possible, while still matching by birth-cohort, to ensure that they were less likely to become a case. Despite selecting the most distantly related individuals possible, it should be noted that the Case-Control cohort contains related individuals, and these relationships for the purpose of association testing must be taken into account. For the Nuclear Family cohort, we selected 39 parent/affected offspring trios, with the non-transmitted alleles from the two parents serving as controls, and 167 female sibships each containing at least one affected and one unaffected sibling, with the unaffected sibling(s) serving as control(s). Blood samples were collected on all subjects and all individuals gave informed consent. This study was approved by the University of Utah Institutional Review Board.

We analyzed associations of the two *NBS1 *tSNPs with breast cancer status as well as for a subgroup analysis of all breast cancer cases, age-at-diagnosis. We examined each tSNP independently and in multi-locus combinations as composite genotype tests and haplotype-based analyses. The empirical null distributions and p-values were determined from a sample size of 2,000 null configurations. For the single locus analyses, the allele frequency estimation method 'GeneCounter' was used. For the composite genotype and haplotype analyses, for which PedGenie requires haplotype frequencies and recombination fractions between loci, haplotype frequencies were determined using 94 unrelated breast cancer cases (n = 47) and controls (n = 47) selected from the 139 breast cancer pedigrees using an expectation-maximization algorithm [42]. The recombination fraction was set to zero for the gene-drop as the two *NBS1 *tSNPs are only ~16 kb apart.

For haplotype analyses, PedGenie requires phase information. We inferred phase information for all genotyped subjects, ignoring relationships, using an expectation maximization (EM) algorithm [42]. Only haplotypes that could be assigned to an individual with >80% probability were accepted. As the EM algorithm [42] is designed for unrelated individuals, all assigned haplotypes were checked for segregation within a family wherever possible. Haplotypes that were incompatible within a family were set to zero.

## Results

### Validation of statistics with simulated independent data

Table 1 shows the results comparing PedGenie *p*-values to those derived from the standard distributions for simulated data. Using both independent case-control and nuclear family data, the empirical *p*-value results from PedGenie compared well to results from the standard distribution, illustrating that for independent samples, the Monte Carlo simulation employed by PedGenie is valid. Several *p*-values from PedGenie were estimated precisely to 3 decimal places when compared to the standard distribution, the majority were within 0.001, and none were significantly different from that expected compared to the standard distribution.

### Validation of inheritance with simulated pedigree data

Table 2 shows the results comparing the Chi-square trend test (weighted to provide dominant and recessive tests) from PedGenie to the Slager and Schaid Armitage trend method [20] using GAW12 simulated pedigree data. Overall, PedGenie compared well. Again, a *p*-value to 3 decimal places matched precisely between PedGenie and the Slager and Schaid method, and other results were not significantly different. There was a tendency for the significance results from PedGenie to be more conservative. The Armitage test for trend asymptotically follows a Chi-squared distribution with one degree of freedom and some appreciable discrepancies between the Armitage statistic significance probabilities, assuming the asymptotic distribution, and the exact Binomial probabilities have been noted for the lower end of the distribution [38]. Overall, these results illustrate that PedGenie accounts for relationships between individuals in an appropriate manner.

### Testing of the NBS1 gene and breast cancer

Table 3 shows the characteristics of the breast cancer real data set composed of 1,034 individuals: 462 cases and 572 controls. In the Case-Control cohort, complete genotype data was available for 231 cases and 235 controls. For the Nuclear Family subset, there were 39 trios including 50 breast cancer cases, as some parents were also cases, and 167 unique female sibships containing 181 cases and 275 controls. The Case-Control cohort was diagnosed at a slightly older age than the Nuclear family cohort.

Table 4 reports all of the statistics that can be run by PedGenie for each of the two *NBS1 *tSNPs tested separately. We observed nominally significant results for rs709816 under a dominant model for the Chi-square (p = 0.015) and odds ratio tests (OR = 1.61, 95% CI: (1.10, 2.35), p = 0.019). In particular, it can be seen that the majority of the difference observed in the dominant model for breast cancer status was due to heterozygous individuals compared to the homozygous wild-type individuals (odds ratio = 1.77, 95% CI: (1.16, 2.72), p = 0.006). No significant results were observed for the age-at-diagnosis data and no significant results were observed using the TDT based tests with Nuclear Family data for either affected status or age-at-diagnosis phenotypes.

The composite genotype results for the *NBS1 *tSNPs are reported in Table 5 using the Case-Control cohort. None of the various combinations of inheritance models across the two loci achieved significance.

Table 6 illustrates the haplotype results from PedGenie for *NBS1*, again using the Case-Control cohort. Comparing all haplotypes to the most common haplotype (1–1) for breast cancer status, a single haplotype (1–2) was found to be nominally significant (p = 0.03). No haplotypes were found to be significant for age-at-diagnosis.

## Discussion

This paper presents an approach and software (PedGenie) for association testing that incorporates family data of any size or structure, allowing for a mixture of structures from singletons to large extended pedigrees and genealogies to be analyzed together. The approach can be used to generate valid tests for any statistic. It is intuitively simple to understand and efficiently utilizes all information available within a family resource.

Another advantage of PedGenie is that it does not require a quantitative trait or the test statistic itself to have a known distribution. Thus, many of the inflexibilities of traditional tests can be avoided. For instance, the QTDT software requires that the quantitative trait be normally distributed. In contrast, using PedGenie to calculate the empirical *p*-value for an observed QTDT statistic does not necessitate this assumption. The traditional TDT and sib-TDT require that a single trio or a single-sibship be selected from each pedigree to be valid tests of both linkage and association. In contrast, PedGenie, which is able to test multiple nuclear families at a time within a pedigree, uses all available data in the pedigree.

Here we have demonstrated PedGenie's ability to perform valid classical tests of dichotomous outcome as well as tests involving quantitative trait data. Multiallelic markers can be analyzed, with user-defined groups, if desired. Tests of composite genotype data and haplotype analyses can all be performed, including composite genotype and haplotype TDT statistics.

We illustrated PedGenie's validity by examining its ability to simulate correctly from the empirical null distribution a *p*-value that corresponds well to the standard distribution for a particular statistical test. We have also shown that PedGenie accounts for relationships within a pedigree similar to an exact method. Finally, we illustrated PedGenie's functionality and ability to assess associations between the outcome variables breast cancer status and breast cancer age-at-diagnosis with tSNPs in the *NBS1 *gene in some large Utah high-risk breast cancer families. In this real data, we found a nominally significant association for a common variant in the *NBS1 *gene (rs709816, *p *= 0.019) indicating a potential role in breast cancer status, particularly for heterozygous carriers of this tSNP (*p *= 0.006). The magnitude of the association results when variants were considered separately versus in combination (unphased multi-locus or phased haplotype data) was strongest for the tSNP rs709816 analyzed separately. For the two *NBS1 *tSNPs analyzed, we observed moderate linkage disequilibrium accounting for minor allele frequencies (D' = 0.775) and low absolute LD (D = 0.128), indicating that the haplotype results are likely driven by rs709816. The decreased significance observed for the composite genotype and haplotype analyses further corroborated these results. As the tSNP rs709816 results in a synonymous (silent) mutation in exon 7 of the *NBS1 *gene, the most likely causal allele for the association observed between the *NBS1 *gene and breast cancer is in linkage disequilibrium with this particular SNP.

Prior evidence for the involvement of the *NBS1 *gene with breast cancer is limited. Homozygous truncating germline mutations in the *NBS1 *gene (~90% due to 657del5 mutation on exon 6) result in the Nijmegen chromosomal breakage syndrome (NBS), a rare autosomal recessive disorder that includes an increased susceptibility to lymphoid malignancy. Heterozygous carriers of the 657del5 mutation have been suggested in a series of Polish case-control studies to be associated with an increased risk of breast cancer [43-45], although the *NBS1 *657del5 Slavic founder mutation is relatively rare even in Poland [44,45]. Several other distinct *NBS1 *mutations of non-Slavic origin have been described [46-48] but their association with breast cancer has not been studied in depth. A limited number of common SNP variants across the *NBS1 *gene have been studied for their association with breast cancer [49] but no significant differences between breast cancer cases and controls were observed. In our study, we systematically identified tagging-SNPs for the *NBS1 *gene, tested the tSNPs in high-risk breast cancer families that are relatively homogeneous, and accounted for all known relationships in our statistical tests. The *NBS1 *protein is involved in variety of processes including sensing DNA damage, DNA double strand break repair, telomere maintenance, and cell cycle checkpoint regulation [50,51], hence it is a likely candidate for a breast cancer susceptibility gene. It follows that replication of our results and further studies involving other *NBS1 *variants are essential.

Empirical methods are often criticized for the computation time required to compute an empirical *p*-value; however, this does not seem to be a serious problem with PedGenie. Analysis of all our 139 pedigrees (3,761 total individuals) for the Case-Control cohort for two tSNPs tested separately, 13 tests, and 2000 simulations each using the allele frequency estimation method 'all' required 4 minutes, 30 seconds using a Dell Precision 650 n 2 × 2.8 GHz Xeon workstation. The same analysis using the allele frequency estimation of 'GeneCountAllele' took 24 minutes, 8 seconds. The majority of analysis time required for PedGenie is spent generating the null genotypic configurations.

Family-based association methods have the disadvantage of being less efficient than the traditional case-control study. The TDT test, for example, has an efficiency of 2/3 for father-mother-child trios compared to case-control studies [52] and this increases the cost of a family-based design. This disadvantage argues strongly that new resources for association should concentrate on ascertaining independent individuals. However, for already ascertained resources, as we have here for breast cancer, pedigree-based methods are a reasonable choice and valid methods, such as those described here, are required.

## Conclusion

Our empirical method provides a valid approach to perform association and TDT testing in pedigrees of any size for both qualitative and quantitative data. Our software, PedGenie, which currently implements a broad range of association-based statistics is freely available from our website.

## Availability and requirements

**Project name: **PedGenie version 1.2

**Project home page: **

**Operating system: **Platform independent

**Programming language: **Java 1.4 and Java 1.5 (for GeneCountAllele method)

**Other requirements: **Java 1.5 or higher

**License: **none required

**Any restrictions to use by non-academics: **none

## Abbreviations

tSNP – tagging-single nucleotide polymorphism

TDT – transmission disequilibrium test

IBD – inherited by descent

EM – expectation maximization

LD – linkage disequilibrium

## Authors' contributions

KAB carried out the genotyping for the *NBS1 *gene, performed the statistical analyses, participated in the design of the software, and drafted the manuscript. JW performed the programming of PedGenie and participated in the design of the software. NJC conceived of the methodology, directed the design of the software, and helped to draft the manuscript. All authors read and approved the final manuscript.

## Figures and Tables

**Table 1:** Comparison of PedGenie to the standard distribution using simulated data for independent individuals and nuclear families

			----Odds Ratio----		--- Difference in Means---						
	Chi Square^2^	Chi Square Trend^2^	HET vs WT^2^	HOM vs WT^2^	HET vs WT^2^	HOM vs WT^2^	ANOVA^2^	Trio TDT^2^	Sib TDT^3^	Comb TDT^3^	QTDT^4^
Statistic	0.292	0.288	0.968	0.949	0.149	0.130	0.017	2.463	1.667	1.563	1.77
*p*-value standard distribution	0.864	0.592	0.632	0.754	0.882	0.896	0.983	0.117	0.096	0.118	0.077
95% CI standard distribution	-	-	0.846–1.107	0.682–1.320	-	-	-	-	-	-	-
Mean empirical *p*-value (SD)	0.865 (0.008)	0.591 (0.012)	0.633 (0.021)	0.749 (0.021)	0.882 (0.007)	0.896 (0.007)	0.983 (0.003)	0.117 (0.008)	0.087 (0.006)	0.118 (0.007)	0.078 (0.006)
P value: interquartile range	0.860–0.870	0.583–0.599	0.619–0.648	0.735–0.763	0.878–0.886	0.891–0.902	0.981–0.985	0.112–0.122	0.083–0.091	0.113–0.123	0.074–0.082
95% CI Empirical (Mean values)	-	-	0.846–1.107	0.685–1.314	-	-	-	-	-	-	-

^1^For each statistical test, PedGenie was run 1,000 times to compute the average, standard deviation, and interquartile distance (1^st ^and 3^rd ^quartile values). The standard distribution is the standard distribution used for the particular statistical test (e.g., the *p*-value reported for the Chi-square test is from the Chi-square distribution). HOM = homozygous for minor allele, HET = heterozygous, WT = wild type.

^2^Test run using 2,000 independent, unrelated cases and 2000 independent, unrelated controls

^3^Test run using 4,000 independent nuclear families, composed of either trios (2 parents and one affected offspring), sib-pairs (one affected and one unaffected sib), or for the combined analysis, a combination of both trios (2,000) and sib-pairs (2,000).

^4^QTDT analysis run using method of Monks *et al*.[11]

**Table 2:** Results for PedGenie Chi-square statistic and Slager and Schaid Armitage test for trend using GAW12 data^1^

**SNP ID**	**MOI**^2^	**Minor Allele Freq**	**D'**^**3**^	**r**^**2**^	**PedGenie Chi-Square *p*-value: Mean (SD) [Interquartile distance]**	**Slager & Schaid Trend Test *p*-value**
5782	D	0.092	–	–	<0.001^4^	2.06E-13
5782	R	0.092	–	–	0.232 (0.010) [0.225–0.239]	0.213
5007	D	0.093	1.00	1.00	<0.001^4^	3.42E-13
5007	R	0.093	1.00	1.00	0.232 (0.010) [0.225–0.238]	0.213
4848	D	0.40	1.00	0.064	0.030 (0.004) [0.027–0.033]	0.030
4848	R	0.40	1.00	0.064	0.693 (0.010) [0.687–0.700]	0.688
11146	D	0.029	0.84	0.003	0.219 (0.009) [0.213–0.225]	0.212
11146	R	0.029	0.84	0.003	-^5^	-^5^

^1^PedGenie results for the Chi-Square test averaged over 1000 runs are compared to the Slager and Schaid Armitage test for trend [20] using prior coefficient of kinship probabilities in the correlation matrix. The 'answer' in this simulated data set is SNP 5782, dominant model.

^2^Mode of inheritance. D = dominant R = recessive

^3^D' and r^2^: linkage disequilibrium measures, calculated between SNP 5782 and each subsequent SNP

^4^The significance threshold of PedGenie is limited by the number of simulations used to create the empirical null distribution (i.e., 2,000). For this result, all statistics based on observed values were less than the statistics based on simulated values.

^5^Result could not be calculated due to sparse data

**Table 3:** Characteristics of Breast Cancer Cohorts selected from 139 high-risk breast cancer pedigrees

	Nuclear Family Cohort^1^				Case-Control Cohort^2^	
	Trio Cases	Trio Controls	Sib Cases	Sib Controls	Cases	Controls
No. with complete genotype data	50	62	181	275	231	235
						
Mean age at diagnosis (SD)	50.2 (12.3)	NA	51.7 (16.2)	NA	58.3 (13.0)	NA
						
Mean age (SD) of controls at time of study	NA	84.5 (9.4)	NA	68.8 (15.0)	NA	74.8 (14.7)

^1^The Nuclear Family cohort consists of 39 trios (i.e., two parents and their affected daughter) and 167 unique female sibships. Some of the parents of the Trios were also breast cancer cases. Some sibships contained more than one female breast cancer case and more than one female control sibling.

^2^The Case-Control cohort originally was composed of 236 breast cancer cases matched to 236 controls. Individuals in this cohort are also related to one another. Genotyping could not be completed for five cases and one control.

**Table 4:** Association of each *NBS1 *tSNP with breast cancer status and age-at-diagnosis of breast cancer

a. Case-Control cohort					
		**rs12680687**		**rs709816**	
	
Weight	Statistic	Statistic (Empirical 95% CI)	*p*	Statistic (Empirical 95% CI)	*p*

Dominant	Chi-Square^1^	1.42	0.26	**6.35**	**0.015**
	Odds Ratio^1^	1.25 (0.85, 1.83)	0.25	**1.61 (1.10, 2.35)**	**0.019**
	Difference in Means^2^	0.68	0.51	-0.11	0.91
Recessive	Chi-Square^1^	2.48	0.13	0.076	0.78
	Odds Ratio^1^	0.56 (0.24, 1.31)	0.18	0.93 (0.51, 1.68)	0.78
	Difference in Means^2^	-0.57	0.60	-0.47	0.62
Additive	Chi-Square Trend^1^	0.086	0.80	2.73	0.10
	Odds Ratio				
	HET vs. WT^1^	1.43 (0.95, 2.16)	0.079	**1.77 (1.16, 2.72) **	**0.006**
	HOM vs. WT^1^	0.64 (0.27, 1.49)	0.31	1.22 (0.68, 2.19)	0.53
	Means				
	HET vs. WT^2^	0.80	0.44	0.037	0.97
	HOM vs. WT^2^	-0.34	0.74	-0.42	0.66
	ANOVA^2^	0.41	0.68	0.10	0.91
Allele tests	Chi-Square^1^	0.09	0.79	2.99	0.084
	Odds Ratio^1^	1.047 (0.77, 1.42)	0.78	1.27 (0.97, 1.67)	0.088
	Means^2^	-8.19	0.31	-4.05	0.61

b. Nuclear Family cohort					
		**rs12680687**		**rs709816**	
		
		Statistic	*p*	Statistic	*p*
	Trio TDT^1^	0.15	0.72	0.04	0.83
	Sib TDT^1^	1.52	0.095	0.45	0.58
	Combined TDT^1^	-1.20	0.23	-0.41	0.70
	Trio QTDT^2^	-^3^	-	-^3^	-
	Combined QTDT^2^	0.92	0.37	0.69	0.51

^1^Breast cancer status was used as the dependent variable

^2^Age-at-diagnosis was used as the dependent variable

^3^The Trio QTDT requires a minimum of 30 trio sets that contain at least one heterozygous parent, complete genotype data on the parents, and age-at-diagnosis of the affected offspring. We had only 25 probands that met all of these criteria; hence the test could not be run.

**Table 5:** Associations of *NBS1 *with breast cancer status and age-at-diagnosis using a composite genotype analysis in the Case-Control cohort^1^.

	Breast Cancer Status		Age-at-Diagnosis	
Model	Odds ratio (Empirical 95% CI)	*p*	Means test	*p*
Dom-Dom	1.23 (0.85, 1.79)	0.28	0.30	0.77
Rec-Rec	0.49 (0.18, 1.29)	0.13	0.005	0.99
Rec-Dom	0.56 (0.25, 1.27)	0.16	-0.59	0.55
Dom-Rec	0.90 (0.48, 1.71)	0.77	-0.79	0.43

^1^Results for this table are derived from requiring specific inheritance models for the two tSNPs for *NBS1 *(rs12680687 and rs709816). Dom = dominant, Rec = recessive.

**Table 6:** Associations of *NBS1 *with breast cancer status and age-at-diagnosis using a haplotype analysis in the Case-Control cohort

		Breast Cancer Status			Age-at-Diagnosis	
Haplotype	Freq^1^	Odds ratio	*p*	Empirical 95% CI	Means test	*p*
1–1	0.63	Reference	-	-	Reference	-
2–2	0.21	1.25	0.25	(0.86, 1.82)	0.31	0.76
1–2	0.12	**1.73**	**0.03**	**(1.04, 2.99)**	-1.079	0.28
2–1	0.037	1.13	0.74	(0.53, 2.39)	-0.10	0.92
